# Genetic characterization of *Theileria equi* infecting horses in North America: evidence for a limited source of U.S. introductions

**DOI:** 10.1186/1756-3305-6-35

**Published:** 2013-02-11

**Authors:** Carina M Hall, Joseph D Busch, Glen A Scoles, Kristina A Palma-Cagle, Massaro W Ueti, Lowell S Kappmeyer, David M Wagner

**Affiliations:** 1 Center for Microbial Genetics and Genomics, Northern Arizona University, 1298 S Knoles Drive, 86011, Flagstaff, AZ, USA; 2 USDA-ARS, Animal Disease Research Unit, 3003 Animal Disease Biotechnology Facility, Washington State University, 99164, Pullman, WA, USA

**Keywords:** *Babesia equi*, *Theileria equi*, Equine piroplasmosis, 18S rRNA gene, Microsatellite, Population genetics

## Abstract

**Background:**

*Theileria equi* is a tick-borne apicomplexan hemoparasite that causes equine piroplasmosis. This parasite has a worldwide distribution but the United States was considered to be free of this disease until recently.

**Methods:**

We used samples from 37 horses to determine genetic relationships among North American *T. equi* using the 18S rRNA gene and microsatellites. We developed a DNA fingerprinting panel of 18 microsatellite markers using the first complete genome sequence of *T. equi*.

**Results:**

A maximum parsimony analysis of 18S rRNA sequences grouped the samples into two major clades. The first clade (*n* = 36) revealed a high degree of nucleotide similarity in U.S. *T. equi*, with just 0–2 single nucleotide polymorphisms (SNPs) among samples. The remaining sample fell into a second clade that was genetically divergent (48 SNPs) from the other U.S. samples. This sample was collected at the Texas border, but may have originated in Mexico. We genotyped *T. equi* from the U.S. using microsatellite markers and found a moderate amount of genetic diversity (2–8 alleles per locus). The field samples were mostly from a 2009 Texas outbreak (*n* = 22) although samples from five other states were also included in this study. Using Weir and Cockerham’s *F*_ST_ estimator (*θ*) we found strong population differentiation of the Texas and Georgia subpopulations (*θ* = 0.414), which was supported by a neighbor-joining tree created with predominant single haplotypes. Single-clone infections were found in 27 of the 37 samples (73%), allowing us to identify 15 unique genotypes.

**Conclusions:**

The placement of most *T. equi* into one monophyletic clade by 18S is suggestive of a limited source of introduction into the U.S. When applied to a broader cross section of worldwide samples, these molecular tools should improve source tracking of *T. equi* outbreaks and may help prevent the spread of this tick-borne parasite.

## Background

The apicomplexan protozoan *Theileria equi* (also known as *Babesia equi*) causes equine piroplasmosis (EP) and is a growing threat to the horse industry [1-3]. *T. equi* is an obligate intracellular parasite that requires a tick host for sexual reproduction and an equine host for asexual reproduction during a haploid phase [2]. *T. equi* can be naturally transmitted by ticks of the family Ixodidae [4] and, in addition to this biological route, *T. equi* has the potential to be transmitted iatrogenically [5]. In fact, iatrogenic transmission is thought to have been the primary mode of transmission in an outbreak in Florida, U.S., in 2008 [6]. Once a horse becomes infected, the parasite undergoes asexual reproduction within erythrocytes; high parasitemia is found during this acute phase of infection [7]. If the horse overcomes the acute infection it will typically carry a life-long persistent infection that is usually asymptomatic [8]. Due to the low parasitemia that is characteristic of a persistent infection the parasite is not detectable by microscopic examination of blood smears. However, infections can be detected by serology and PCR. Although asymptomatic horses have low parasitemia, transmission of *T. equi* can still occur either iatrogenically, or when competent tick vectors feed on these horses [2,9]. Thus, asymptomatic persistently infected carriers can serve as reservoirs of infection, which is one of the challenges for controlling the spread of this parasite.

When naïve horses are parasitized by *T. equi* the infection can cause a range of disease symptoms up to and including death. Although this parasite is less prevalent in countries such as the U.S., Australia, England and Canada, even with transport regulations there is a potential for spread from infected horses or ticks from endemic regions [10]. The U.S. was thought to be free of EP since 1988 as the result of a twelve million dollar, 25 year-long eradication campaign that began in 1962 [6]. However, since 2008 numerous cases of *T. equi* infection have been identified in the U.S. in California, Colorado, Florida, Georgia, Oklahoma and Texas. These are most likely the outcome of importing asymptomatic horses that produced negative results during mandatory screening procedures [6,11,12]. A large outbreak in Texas caused alarm when positive horses from 16 states were epidemiologically traced back to a single source ranch [11,12]. With over 9.2 million horses in the U.S., a widespread outbreak could have a large financial impact on the $39 billion horse industry [13]. Due to concerns about importing *T. equi* into non-endemic regions, the World Organization for Animal Health (OIE) and U.S. Department of Agriculture (USDA) implemented a mandatory screening process for international movement of horses. When horses are imported into the U.S. they are screened with a serological assay to ensure they are free of *T. equi*. Prior to 2005 the complement fixation test (CFT) was used as the regulatory test of choice, but since that time import testing has included a competitive enzyme-linked immunosorbent assay (cELISA) [14]. DNA-based methods have also been developed for screening horses and these may be more sensitive for detecting early infection, or very low-level parasitemia [15].

Molecular genetic tools provide a powerful means for detecting and tracking the spread of cryptic pathogens and parasites such as *Plasmodium falciparum* and *P. vivax*[16-19]. Although molecular tools have been developed for *T. equi* to provide detection (*ema-1* gene) and broad phylogenetic classification (18S rRNA gene) [20-22], to date there is no system available for genotyping with microsatellite markers. These highly variable markers provide fine-scale resolution for epidemiological tracking, evaluating genetic structure and identifying single versus mixed infections of haploid clones. Mixed infections are an important factor in protozoan diseases and can lead to increased virulence compared to single clone infections [23]. The use of both neutral microsatellite markers and gene sequences such as the 18S rRNA gene greatly enhances the ability to conduct population genetic analyses on pathogens [24].

The goal of this study was to understand genetic diversity among *T. equi* samples from North America. First, we examined full-length 18S rRNA gene sequences to provide broad phylogenetic groupings for all samples into the four previously described clades of *T. equi*[21,25]. Next, we developed a panel of 18 microsatellite markers to provide finer-scale resolution of these genetic relationships. The first whole genome sequence of *T. equi*[26] provided the foundation for the development of highly variable microsatellite markers for *T. equi*. This genotyping panel was used to determine the genetic diversity of *T. equi* strains in the recent Texas outbreak and to trace back samples from potential sources of *T. equi* in the southern U.S. Both types of molecular data provided insight into the number of genetic lineages in the U.S. and the amount of genetic diversity within subpopulations.

## Methods

### *T. equi* DNA samples and preparation

The *T. equi* genomic DNA (gDNA) samples used in this study were collected by the USDA-ARS, Animal Disease Research Unit in Pullman, WA from routine screenings and as part of the response to recent outbreaks. We used a set of 38 samples from six states to maximize geographic diversity within the U.S. as much as possible (Table 1). Later, two samples were removed due to low data quality; Te0021 was excluded from 18S analysis and Te0044 was excluded from the microsatellite analysis. A major focus was on samples from the Texas outbreak, which affected several hundred horses in 2009 [11]. Two isolates were derived from *Amblyomma cajennense* (Te0002) and *Dermacentor variabilis* (Te0035) ticks collected from multiple horses during the Texas outbreak [11]. These ticks were fed on uninfected horses at the USDA lab and once infections were confirmed the isolates were preserved as frozen blood stabilates, as previously described [11]. Another isolate, Te0042, was collected during the 2008 Florida outbreak [6]. Our positive control was a lab strain, Te0003, which originated from a Florida outbreak in the 1970s and was the same strain used for the first *T. equi* whole genome sequence (WGS) [26]. All but one of the samples were from infected U.S. horses; Te0044 was collected from a stray horse captured crossing the U.S.-Mexico border near Eagle Pass, TX. This horse tested as a weak seropositive for *T. equi* using the official cELISA regulatory test, but yielded negative results using nested PCR targeting *ema-1*. Infection was confirmed in this horse by passaging 100 mL of whole blood into a naïve splenectomized horse via intravenous inoculation. The transmission was successful and at the peak parasitemia (10% parasitized erythrocytes) blood samples were collected for genotyping. Genomic DNA was isolated during acute infection and nested PCR targeting *ema-1* again yielded negative results. Sequencing of the 18S rDNA [GenBank:JQ390047] demonstrated 52 bp divergence from the 18S gene found in the Florida *T. equi* whole genome sequence.

**Table 1:** ***T. equi *samples extracted from horse blood for use in this study**

**NAU ID**	**Location**	**County**	**Year collected**	**Details**	**Passage details of sample**	**MSAT**	18S group**
Te0002*	TX	Kleberg	2010	horse infected with *Amblyomma cajennense* ticks collected from 73 TX horses	tick passage from field (from multiple hosts)	yes	A
Te0003*	FL		2009	lab strain originally from 1970s outbreak (whole genome sequence)	multiple needle passages	yes	A
Te0004	TX	Kleberg	2009	mare from index ranch breeding herd	field blood (direct from horse)	yes	A
Te0005	TX	Kleberg	2009	mare from index ranch breeding herd	field blood (direct from horse)	yes	A
Te0006	TX	Kleberg	2009	mare from index ranch breeding herd	field blood (direct from horse)	yes	A
Te0007	TX	Kleberg	2009	mare from index ranch breeding herd	field blood (direct from horse)	yes	A
Te0008	TX	Kleberg	2009	mare from index ranch breeding herd	field blood (direct from horse)	yes	A
Te0009	TX	Kleberg	2009	mare from index ranch breeding herd	field blood (direct from horse)	yes	A
Te0010	TX	Kleberg	2009	mare from index ranch breeding herd	field blood (direct from horse)	yes	A
Te0011	TX	Kleberg	2009	mare from index ranch breeding herd	field blood (direct from horse)	yes	A
Te0012	TX	Kleberg	2009	mare from index ranch breeding herd	field blood (direct from horse)	yes	A
Te0013	TX	Kleberg	2009	mare from index ranch breeding herd	field blood (direct from horse)	yes	A
Te0014	TX	Kleberg	2009	mare from index ranch breeding herd	field blood (direct from horse)	yes	A
Te0015	TX	Kleberg	2009	mare from index ranch breeding herd	field blood (direct from horse)	yes	A
Te0016	TX	Kleberg	2009	mare from index ranch breeding herd	field blood (direct from horse)	yes	A
Te0017	TX	Kleberg	2009	mare from index ranch breeding herd	field blood (direct from horse)	yes	A
Te0018*	TX		2009	retired racehorse-epidemiologic trace out, pastured with Be0019, Be0020	needle passage from field	yes	A
Te0019*	TX	Kleberg	2009	pastured with Be0018-epidemiologic trace out	needle passage from field	yes	A
Te0020*	TX	Kleberg	2009	pastured with Be0018-epidemiologic trace out	needle passage from field	yes	A
Te0021	CA		2010	routine screening, presumed to be unrelated to outbreak	field blood (direct from horse)	yes	no sequence data
Te0022	CA	San Diego	2010	routine screening, presumed to be unrelated to outbreak	field blood (direct from horse)	yes	A
Te0023	GA		2010	routine screening, presumed to be unrelated to outbreak	field blood (direct from horse)	yes	A
Te0024	GA		2010	routine screening, presumed to be unrelated to outbreak	field blood (direct from horse)	yes	A
Te0025	GA		2010	routine screening, presumed to be unrelated to outbreak	field blood (direct from horse)	yes	A
Te0026	GA		2010	routine screening, presumed to be unrelated to outbreak	field blood (direct from horse)	yes	A
Te0027	GA		2010	routine screening, presumed to be unrelated to outbreak	field blood (direct from horse)	yes	A
Te0028	GA		2010	routine screening, presumed to be unrelated to outbreak	field blood (direct from horse)	yes	A
Te0029	GA		2010	routine screening, presumed to be unrelated to outbreak	field blood (direct from horse)	yes	A
Te0033	OK		2010	routine screening, presumed to be unrelated to outbreak	field blood (direct from horse)	yes	A
Te0034	CO		2010	routine screening, presumed to be unrelated to outbreak	field blood (direct from horse)	yes	A
Te0035*	TX		2009	horse infected with *Dermacentor variabilis* ticks collected from 17 TX horses	tick passage from field (from multiple hosts)	yes	A
Te0039*	TX	Cameron	2011	index ranch-treatment study	needle passage from field	yes	A
Te0040	TX	Kleberg	2011	index ranch-housed at USDA	field blood (direct from horse)	yes	A
Te0041	TX	Kleberg	2011	index ranch-housed at USDA	field blood (direct from horse)	yes	A
Te0042*	FL		2008	2008 Florida outbreak	needle passage from field	yes	A
Te0045	TX	Kleberg	2011	index ranch-treatment study	field blood (direct from horse)	yes	A
Te0046	TX	Kleberg	2011	index ranch-treatment study	field blood (direct from horse)	yes	A
Te0044*	U.S.-MX border	Maverick	2011	stray intercepted crossing TX-Mexico border	needle passage from field	no (amplification at only 7/18 MSAT loci)	C

*Isolate, which refers to strain types derived from infections that were reproduced in the lab and available as frozen blood stabilates for future study.

**Figure 1:**
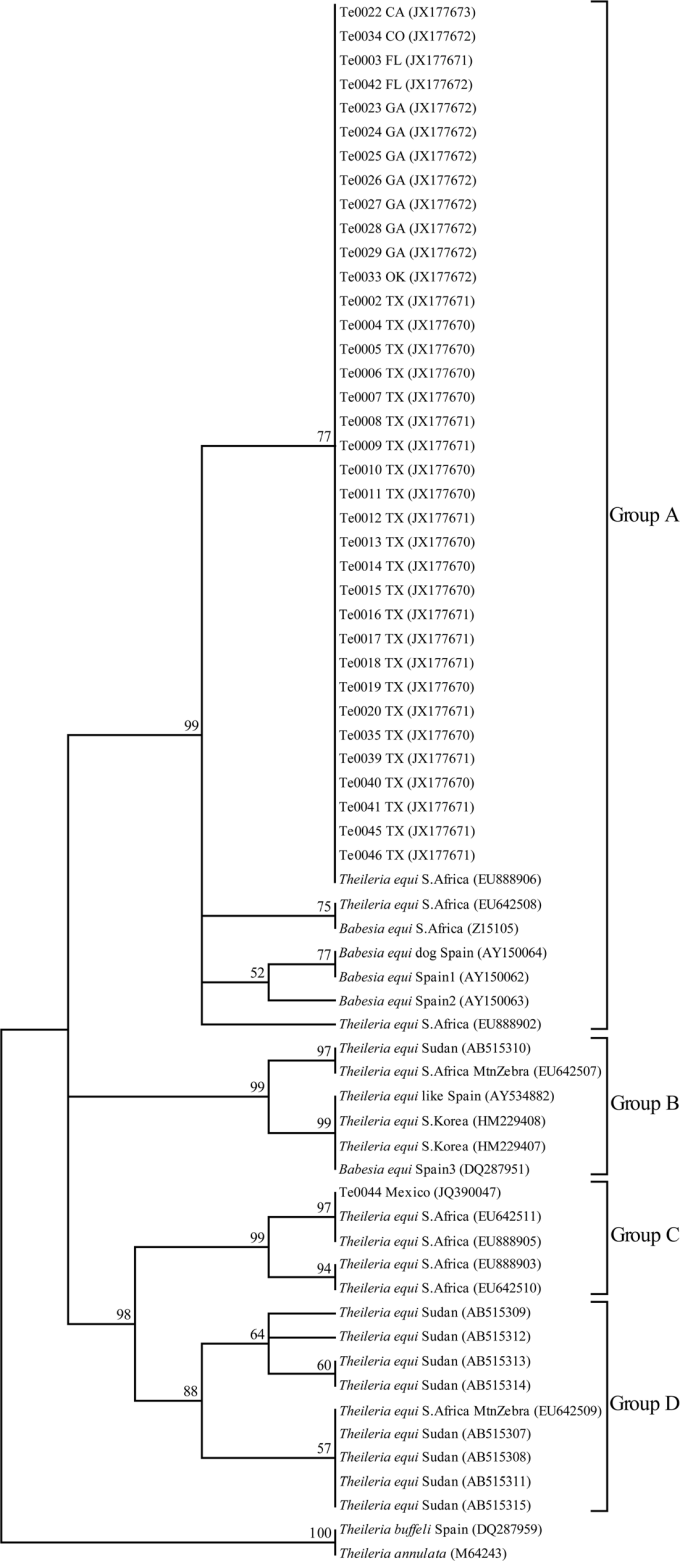
**Maximum Parsimony tree of *T. equi *18S rRNA gene sequences with 1,000 bootstrap replicates.** The tree is rooted with *Theileria annulata* and *T. buffeli*.

**As previously defined by Bhoora *et al.*[21], 2009 and illustrated in Figure 1.

DNA from *T. equi*-infected horse blood was prepped with either the Qiagen® DNeasy Blood & Tissue Kit or the Qiagen® Gentra PureGene Blood Kit (Valencia, CA). Before extraction, horse blood was centrifuged for 15 minutes at 2,000 × g to pellet red blood cells and the white blood cell layer was removed to minimize the amount of horse DNA in the final preparation. To obtain a higher yield of parasite DNA from some of the persistently infected horses that were sampled, anti-coagulated whole blood was transferred to uninfected horses at the USDA lab. Blood was collected from the recipient for DNA extraction at peak parasitemia. The volume of blood transferred from persistently infected horses ranged between 10–60 mL; the goal was to transfer a sufficient amount of blood to ensure that the full diversity of *T. equi* from the field horse was transferred to the naïve uninfected horse. All animals used in these studies were handled according to protocols approved by the University of Idaho Institutional Animal Care and Use Committee (protocol #2010-54).

All gDNA samples were processed with whole genome amplifications (WGAs) using Qiagen’s REPLI-g® Mini Kit (Valencia, CA) to increase the limited amount of *T. equi* DNA present in some samples. The manufacturer’s WGA protocol was followed, using 1 μL of gDNA as template. The WGA procedure was also performed on samples with sufficient parasite DNA to insure that all samples were treated the same. In the case of single clone infections a WGA had no effect on the results, because genotypes were the same whether WGA or gDNA was used as template (data not shown). Conversely, the WGAs of mixed clone infections sometimes resulted in different clone ratios due to stochastic bias in the WGA process. To ensure adequate and unbiased representation of the entire genome, three independent WGA replicates were run for each *T. equi* sample. Each WGA replicate was validated using a PCR assay targeting the *ema-1* gene [22]. The published assay was converted into a qPCR assay using SYBR® Green (Invitrogen, Grand Island, NY). WGAs were considered valid if they showed amplification with a cycle time of 30 cycles or less (C_t_ ≤ 30). Validated WGA replicates were pooled together to serve as templates for genotyping.

### Sequence diversity of 18S rRNA gene

Primers specific to the 18S rRNA gene of the genus *Babesia* were designed to amplify and sequence most of the 18S region (~1,600 bp). Primers were designed using sequences obtained from GenBank for a variety of species including *B. equi*, *B. gibsoni*, *B. major*, *B. occultans*, 14 *B. bigemina* and 6 *B. bovis* (accession numbers listed in Additional file 1: Table S1). Sequences were aligned in MegAlign (DNASTAR Lasergene9, Madison, WI) and primer melting temperatures and interactions were investigated with Primer Express® Software v2.0 (Applied Biosystems, Carlsbad, CA). These primers were intended to be used with field samples, where non-target DNA can be present, so optimized primers were sent through a Primer-BLAST in GenBank against all prokaryotes and eukaryotes to ensure other eukaryotic organisms (e.g., protozoans, ticks, and equids) would not amplify. We designed two primers for amplification of the 18S fragment to be sequenced: 18S_AllBab_1F (forward, 5′- AGCCATGCATGTCTAAGTACAAGCTTTT-3′) and 18S_AllBab_R3 (reverse, 5′- TCCGAATAATTCACCG GATCACTC-3′). The initial 10 μL PCR contained final concentrations of the following reagents: 1 μL of 10× buffer, 2.5 mM MgCl_2_, 0.2 mM dNTPs, 0.4 μM of each primer, 0.8 units of Platinum® Taq (Invitrogen, Grand Island, NY) and 1 μL of template (undiluted gDNA). The cycle conditions for the initial PCR consisted of 5 min, 95°C; (30 sec, 94°C; 30 sec, 60°C; 75 sec, 72°C) × 40 cycles; 5 min, 72°C; held at 16°C. The 1,591 bp PCR product (2 μL) was visualized on a 1.5% agarose gel using 1 kb ladder (Invitrogen, Grand Island, NY) as a reference to confirm the correct target size and to estimate the dilution needed for cycle sequencing PCR. Water used for no-template controls (NTC’s) and DNA from uninfected horse blood as a negative control confirmed the assay amplified only *T. equi*. To remove excess primers and dNTPs after completion of the PCR, 1.5 μL of Exo-SAP-IT® (USB Corporation, Cleveland, OH) was added to each reaction and incubated for 15 min at 37°C, followed by enzyme deactivation for 15 min at 80°C. Dilutions of the PCR product were made depending on the band intensity from the gel electrophoresis. Bright bands were diluted 1:5 and faint bands were diluted 1:2.

The 18S rRNA gene amplicons of 37 *T. equi* samples were sequenced with the Sanger method. Four internal primers were developed to allow double-coverage sequencing across the entire 1,591 bp amplicon. We used the diluted PCR product as template for BigDye® Terminator v3.1 cycle sequencing (Applied Biosystems, Carlsbad, CA). Every sample required six sequencing reactions with the following primers: AllBab_1F, sequence listed above; All_Babesia_F2, 5′-CAAGTCTGGTGCCAGCAGCC-3′; All_Babesia_F3, 5′-CAAAGTCTTTGGGTTCTGGGGG-3′; All_Babesia_R1, 5′-CCCTACCGTCAAGCTGATGGG-3′; All_Babesia_R2, 5′-ACGAATGCCCCCAACCGT-3′; AllBab_R3, sequence listed above. About half of our samples had poor reads for the outside forward (AllBab_1F) and reverse (AllBab_R3) contigs. In order to overcome this, nested forward and reverse primers (All_Babesia_F1a, 5′- AGCTTTTATATGGTGAAACTGCGAAT-3′; All_Babesia_R3a, 5′- TCACTCGATCGGTAGGAGCGA-3′) were designed internally to the original PCR primers. The conditions for the cycle sequencing were 3 μL 5× sequencing buffer, 1 μL BigDye® v3.1, 1 μM of a single primer and 5 μL of diluted PCR product producing a 10 μL reaction. The cycle conditions for the cycle sequencing consisted of 5 sec, 96°C; 20 sec, 50°C; 4 min, 60°C; (three first steps repeated 25 times) then held at 16°C. An EDTA/ethanol precipitation cleanup was performed on the products before they were sequenced on a 3130xl Sequencer (Applied Biosystems, Carlsbad, CA).

An assembly of sequences for each individual was accomplished in SeqMan (DNASTAR Lasergene9, Madison, WI) and all sequences were edited by visual inspection. The 37 *T. equi* 18S sequences were exported into BioEdit Sequence Alignment Editor (Carlsbad, CA) to align with 28 other *Theileria* 18S rRNA sequences from GenBank, including *Theileria buffeli* and *T. annulata* as outgroup taxa (accession numbers reported in Figure 1). *T. buffeli* and *T. annulata* are appropriate to serve as an outgroup to *T. equi* for the 18S rRNA gene as demonstrated by Bhoora *et al.*[21]. The 65 18S rRNA gene sequences were aligned using Clustal W Multiple Alignment and gaps were visually inspected and adjusted. The sequences were imported into MEGA version 4 [27] where a maximum parsimony (MP) tree was constructed with a bootstrapping method applied. A consensus tree was created, with only bootstrap values >50% reported (Figure 1).

### Identification of *T. equi* microsatellite sequences and primer design

A whole genome sequence of the USDA Florida lab strain (Te0003) was used to discover microsatellite regions within the *T. equi* genome [26]. A variety of repeat sizes were searched, ranging from dinucleotide to hexanucleotide-repeats, using the software Tandem Repeats Finder [28] and MSATCOMMANDER [29]. Over a hundred loci were identified and primer pairs were designed at 75 of these with target sizes <600 bp. Forward and reverse oligonucleotide primers were developed using NetPrimer (Premier Biosoft International, Palo Alto, CA) and SeqBuilder (DNASTAR Lasergene9, Madison, WI). First the optimal melting temperature (T_m_) of each locus was determined by a temperature gradient PCR using the same conditions as a single PCR (Additional file 2: Table S2) except that a T_m_ range of 55-66°C was used. Then the loci were tested for polymorphism on nine Texas samples using single PCR conditions (Additional file 2: Table S2). If the locus proved to be polymorphic, then an external unlabeled primary forward primer was designed for use in a pre-amplification PCR. The pre-amplification PCR was followed by a heminested PCR using the original forward primer; this double-PCR process has been used to genotype a number of parasite species that often yield very low concentrations of DNA from host blood [20,30,31]. The external forward primer was first optimized using a temperature gradient. Then the PCR product from the primary PCR served as the template for the second (heminested) PCR, which used the internal forward primer. All validation PCRs were visualized on 2% agarose gels stained with SYBR® Safe (Invitrogen, Grand Island, NY) using a 100 bp DNA ladder to estimate sizes. After optimization, either 4 or 5 loci for each of the four chromosomes were found to yield robust PCR products yielding a total of 18 loci (Additional file 2: Table S2). The development of loci from all four chromosomes provided well-spaced markers across the whole genome. The internal forward primer from each locus was fluorescently labeled for high-resolution sizing on an AB3730 capillary machine (Applied Biosystems, Carlsbad, CA).

Electrophoresis was conducted on an AB3730 DNA Analyzer using GeneScan™ 1200 LIZ® Size Standard (Applied Biosystems, Carlsbad, CA). Each well contained 13.92 μL of Hi-Di™ Formamide (Applied Biosystems, Carlsbad, CA) with 0.08 μL size standard and 1 μL of diluted PCR product (1/100 for single PCR and 1/200 for heminested PCR). Plates were denatured for five minutes at 95°C and snap-cooled before each AB3730 run. Automatic scoring was accomplished with GeneMapper Software Version 4.0 (Applied Biosystems, Carlsbad, CA) and all calls were confirmed by visual inspection.

### Microsatellite marker amplification strategy

Persistently infected horses carry only small levels of *T. equi* DNA, reflecting the low parasitemia of chronic infections [22,30]. These samples can yield variable PCR amplification and artifacts. Thus, it was critical to develop a reliable workflow that can be applied reproducibly to every sample to reduce genotyping errors and minimize sample to sample variation. We developed a robust 5-step process to provide consistent and repeatable genotyping for *T. equi*:

1. To conserve the original DNA extraction, which often had low levels of target DNA, and increase the amount of starting target copies for PCR, we used WGAs from each sample as template for the initial round of PCRs.

2. After WGA, samples were initially genotyped using only internal primers in a multiplexed PCR (Additional file 2: Table S2 for compatible multiplexes).

3. If multiple alleles were detected at a single marker, the sample was screened again using unamplified gDNA as template and running each locus singly instead of in multiplex reactions. Genomic DNA provides a better representation of the actual allele abundance than WGAs and allowed us to determine the predominant allele (i.e. the one peak with the largest number of relative fluorescence units (rfu)). Multiple alleles were scored if the rfu of minor peaks were ≥25% of the predominant peak [30].

4. When amplification was weak (<200 rfu), the samples were screened using WGA template in a single PCR without multiplexing.

5. If the above workflow still did not detect any alleles at a locus, we then used a single, heminested PCR. We first used WGA as template and if the sample still did not amplify gDNA template was used. This heminested PCR approach has been successfully employed in malaria genotyping for samples with low levels of target DNA [30,32]. We first amplified using the external, unlabeled primers for each locus individually, as described above (Additional file 2: Table S2). Then 1 μL of this primary PCR was used as the template for a heminested PCR, again amplifying each locus separately.

Regardless of whether single or heminested PCR was used, all alleles were confirmed with a second, independent PCR. If two conflicting genotypes were found then a third PCR was conducted and the allele scored twice as being predominant was used for the final data. If three distinct predominant alleles were called then the most common allele of the three PCRs was used as a final datum. Our strategy was designed to be conservative in calling alleles that are truly present (as recommended by Anderson and others [30]); however, we realize this increases the risk of not capturing the full allelic diversity that may be present in some mixed infections. Frequencies for most common alleles were related to a sample’s subpopulation (Texas, Georgia, Florida, etc.). Missing data at a locus was called when four or more attempts were made at amplification. Four horse samples were excluded from analysis because we were unable to amplify multiple loci, which indicated *T. equi* DNA was either too dilute in the sample or the chromosomal targets were simply absent in that particular strain. All PCR runs included water as an NTC and uninfected horse DNA to ensure false positives were not called due to primer interaction or amplification of horse DNA.

### Population analysis

We used population genetic analysis to determine linkage disequilibrium and genetic structure among the populations investigated in this study. As in many studies of apicomplexans, only primary alleles were used for data analysis [16,33-35]. The complete multi-clone genotype was only used to examine the percentage of samples exhibiting mixed clone infections versus single clone infections. To distinguish between a single clone infection with mutation events and a true mixed infection, we defined mixed clone infections as samples that displayed three or more loci with secondary alleles. Small populations (n ≤ 2 samples) were included in tree construction, but excluded from all other marker validation and statistical analyses of population structure. Using the Texas population, we used the primary alleles amplified for each *T. equi* sample to test for linkage disequilibrium between all pairs of loci using 3060 permutations in FSTAT v2.9.3.2 (Goudet, J., 2001; http://www.unil.ch/izea/softwares/fstat.html). Data were imported into FSTAT using the Excel plug-in Microsatellite Excel Toolkit [36]. Population structure was estimated with Weir and Cockerham’s *F*_ST_ estimator (*θ*) calculated in FSTAT. Using the Excel plug-in GenAlexv.6 [37] we calculated the expected heterozygosity (*H*_E_) at the population level. A neighbor-joining (NJ) tree was generated from mean character distances using PAUP ver. 4.0b10 [38]. Confidence for the NJ tree was estimated by bootstrapping with 500 repetitions.

## Results

### Phylogenetics of the *T. equi* 18S rRNA gene

All *T. equi* samples from the U.S. grouped together into a single monophyletic clade based on 18S rRNA gene sequences. The maximum parsimony (MP) tree was created using 116 parsimony informative sites. One of the 387 MP trees based on 18S rRNA gene sequences is shown in Figure 1 (tree length: 248 steps, consistency index: 0.7450, retention index: 0.9393). Separate phylogenetic analyses using MP and neighbor joining produced similar tree topologies with high nodal bootstrap values (Figure 1) and were consistent with two previous *T. equi* studies that used neighbor joining, maximum likelihood and Bayesian inference on the 18S rRNA gene [21,25]. In the current analysis we recovered the four major phylogenetic groups (A, B, C and D) using GenBank sequences previously described (*n* = 28) [21,25] and the new U.S. sequences we describe in this study all (*n* = 36) fell into group A with sequences from South Africa and Spain (Figure 1). Nucleotide variation within group A is encompassed by less than ten single nucleotide polymorphisms (SNPs). Only two of these SNPs were observed in the U.S. sequences resulting in four distinct haplotypes [GenBank:JX177670, JX177671, JX177672, JX177673], which suggests that a small source population is responsible for all recent U.S. outbreaks. All U.S. samples grouped together in a monophyletic clade with one other South African isolate [GenBank: EU888906]. One U.S. sample from California, Te0022 [GenBank: JX177673], shared an identical sequence with this African isolate.

The sample from the U.S.-Mexico border, Te0044 [GenBank:JQ390047], was different from the other U.S. samples by 48 SNPs and was robustly placed in group C identified by Bhoora *et al.*[21]. This isolate is clearly different from the other North American samples. As noted above, the horse from which this isolate originated was weakly seropositive for *T. equi* using the official cELISA regulatory test, but yielded negative results using nested PCR targeting *ema-1*[22]. This isolate was nearly identical to two South African *T. equi* in GenBank [EU642511, EU888905], with only one SNP separating the Mexican sample from these two sequences.

### Genetic diversity

In contrast to the 18S gene sequences, microsatellite repeat markers exhibited a greater amount of genetic diversity among the U.S. *T. equi* samples. Two to eight alleles were found at each microsatellite locus (Additional file 2: Table S2). No linkage disequilibrium was detected in any combination of locus pairs. The expected heterozygosity (*H*_E_) for the Texas population (*n* = 24) was 0.496 with a standard error of 0.044. Identical dominant genotypes were observed for most of the samples from Georgia (*n* = 6), with only one sample differing at a predominant allele and four others differing at one or two secondary alleles. Despite its physical distance from Georgia, the sample from Oklahoma also shared a dominant haplotype with the Georgian samples, although it carried two unique secondary alleles. The two California samples shared alleles at only five markers. The isolate from the U.S.-Mexico border (Te0044) did not amplify well with this marker set. Only 7 of 18 loci amplified reliably, suggesting that only *T. equi* samples from 18S group C may be typed successfully with this subset of markers (Additional file 2: Table S2). Unfortunately, we did not have samples available from groups B and D to test with this DNA fingerprinting system.

**Figure 2:**
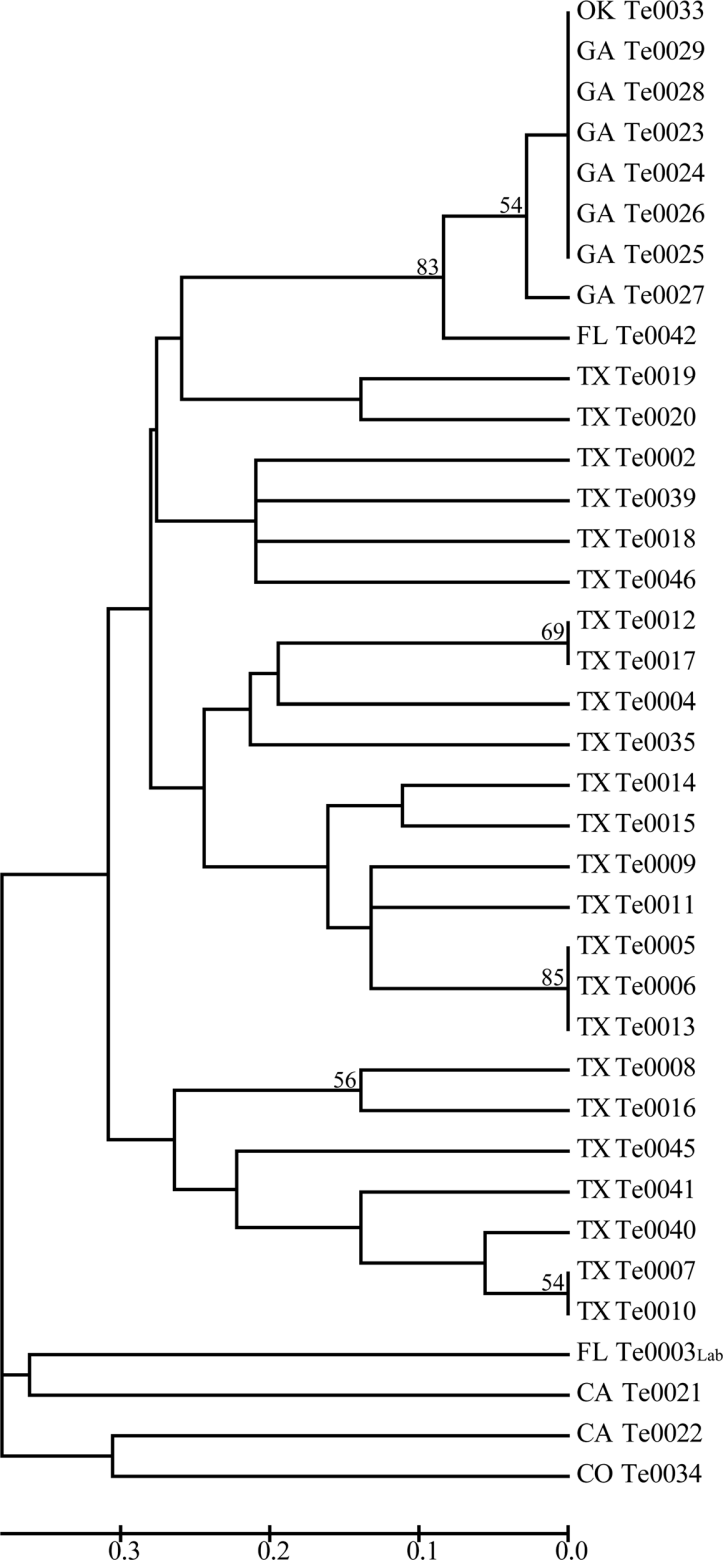
**Mid-point rooted neighbor-joining tree constructed with *T. equi *microsatellite data with 500 bootstrap replicates (only values >50% reported).** Lab = Lab strain, used for WGS.

The set of 18 microsatellite markers revealed geographic structure among U.S. outbreaks of *T. equi*. Texas samples were loosely grouped into a large, diverse lineage (Figure 2). The genetic diversity in Texas may be due to a larger effective population size than other outbreaks and tick-borne transmission resulting in genetic recombination of *T. equi*. In contrast, Georgian samples separated into a separate subpopulation distinct from the Texas group. The Georgia clade consists mainly of a group of samples (*n* = 7) that share the same dominant haplotype. In addition to Georgia, this clade also includes a sample from Oklahoma, suggesting a similar source of infection. The samples from Colorado, California and the Florida lab strain did not cluster strongly with any other outbreaks on the NJ tree. The *F*_ST_ analysis is generally congruent with the NJ tree, since Texas and Georgia populations demonstrate strong differentiation (*θ* = 0.414, 99% CI: Lower = 0.290, Upper = 0.504). Population sizes from all other states were too small for a robust *F*_ST_ analysis.

### Mixed and single infections

Microsatellite analysis revealed that less than one-third of our total sample set (*n* = 10; 27%) contained mixed infections (2–3 clones). We made this estimate based on the greatest number of alleles at any single locus. However, this may be an underestimate if additional alleles were present but not detected. Of the 27 single infection samples, 15 (56%) had unique multilocus haplotypes. Some of the samples with single infections bore evidence of microsatellite mutations, because we occasionally observed additional secondary alleles at 1–2 loci. Two groups of single infection samples that shared haplotypes (Te0012, Te0017 and Te0005, Te0006, Te0013) were collected from mares kept in a common pasture.

## Discussion

Samples of *T. equi* from the recent outbreaks in Texas and Florida, as well as the other U.S. samples, were all assigned to the 18S group A of Bhoora *et al.*[21]. All U.S. samples exhibit very little diversity within this clade, suggesting a limited introduction of a small number of clones into the U.S. This is in strong contrast to the large amount of 18S diversity previously observed in South Africa. The sample from California (Te0022) that shares complete 18S identity with a South African isolate [GenBank:EU888906] may suggest a shared source of introduction for California and the rest of the U.S. Conversely, the *T. equi* strain from the U.S.-Mexico border, Te0044, has a different 18S lineage (group C) compared to the other U.S. samples. This isolate was undetectable with the *ema-1* PCR and, because it has a *T. equi* genotype that is different from other samples, may require the use of a different detection assay to mitigate the risk of moving similar strains of *T. equi* into regions that are free of the parasite [39]. Te0044 is also distinctive because less than half of the microsatellite markers genotyped successfully. Additional development of microsatellite loci may be needed for genotyping all four of the major lineages of *T. equi*.

Our new microsatellite typing system allowed us to examine population structure of *T. equi* with greater resolution than provided by the 18S gene. Although 36 U.S. samples had nearly identical 18S sequences, at least 15 unique clones were detected using microsatellite markers. This finding suggests that since the initial introduction of a limited source into the U.S., *T. equi* populations have developed strong population structure across the spatial scale that we studied. Robust differentiation was observed between Texas and Georgia and this high *F*_ST_ value reflects the pattern also observed in the NJ tree. The greatest amount of genetic diversity was observed among the Texas samples, which might be expected from an outbreak with a greater effective population size [11]. The Texas outbreak was an especially valuable sample set for validating this panel of markers. Epidemiological data associated with each horse helps explain how variation is distributed in the Texas samples and validates identity between certain samples. For instance, the horses that yielded isolates with identical haplotypes, represented in the three clades observed in Figure 2, were mares that shared the same pasture. Therefore, it is highly likely that these horses were infected from a common source or passed the infection from one to another. It appears that transmission during the Texas outbreak was the result of tick-borne transmission; both *Amblyomma cajennense* and *Dermacentor variabilis*, as well as other tick species, may have been involved in transmission of *T. equi* to 292 of the 360 horses on the ranch at the center of the outbreak [11]. Some of the genetic diversity observed among the Texas outbreak samples could be due to recombination during sexual stage development in the tick gut, or by infection from multiple ticks that carried different *T. equi* clones. Additionally, the Texas samples might have been the result of a founding population of clones that carried a large amount of microsatellite variation.

The other U.S. samples differed in their level of genetic variation, which allows us to make inferences about the relatedness of these samples. The two California *T. equi* appeared to be quite distinct from samples from the other states and may have had origins unrelated to that of the Texas outbreak. Unfortunately, we do not have an adequate sampling of the California subpopulation (*n* = 2) to address this question. The 2010 samples from California and Colorado do not appear to be closely related to the samples collected farther east. The similar genotypes found in Georgia, Florida and Oklahoma suggest that there was a shared source of *T. equi* for these populations. Another possibility could be that dominant genotypes in Oklahoma and Georgia represent a highly virulent clone that outcompetes less virulent clones, thus giving the misleading appearance of a shared source of infection.

Our microsatellite typing system will also be valuable for typing isolates obtained from outbreaks resulting from iatrogenic transmission. The largest outbreak of equine piroplasmosis in the U.S. prior to Texas in 2009 was the Florida outbreak of 2008 in which 20 infected horses were identified and euthanized [6]. The epidemiological investigation of the Florida outbreak suggested that iatrogenic transmission was the primary cause. Unfortunately, we had only one isolate from this outbreak (Te0042), but if a larger collection of samples had been available this hypothesis could have been tested using strain typing. Very limited strain diversity would have been expected if transmission were purely mechanical.

A set of three samples illustrates the utility of this genotyping system to address specific epidemiological questions for certain individuals. *T. equi* strains Te0018, Te0019 and Te0020 were collected from horses pastured together. The horse infected with Te0018 was retired after a 20-year racing career, whereas Te0019 and Te0020 were epidemiologically linked to the Texas outbreak ranch. Had one of these horses been the source of infection for the others? The genotyping data suggest not, since Te0018 is distinctly different from Te0019 and Te0020 (Figure 2). Thus, it is highly likely that the sources of infection were unrelated. This trio of samples demonstrates the usefulness of microsatellite markers for tracing back individual samples, which is an important complement to the analysis of population structure for larger outbreaks.

## Conclusions

The introduction of this tick-borne hemoparasite into susceptible regions has the potential to be very disruptive to the U.S. horse industry and should remain a major concern to the horse community and regulatory agencies. The ability of highly variable molecular markers to detect small genetic changes among samples provides a powerful tool for studying the epidemiology of parasitic diseases such as *T. equi*. Selectively neutral microsatellites have been successfully used to determine population structure of other apicomplexan pathogens such as the agent causing malaria, *P. falciparum*[16], bovine theileriosis, *T. annulata*[40], and bovine babesiosis, *B. bovis*[35]. Using our newly designed microsatellite panel we have identified multiple unique clones in recent U.S. outbreaks that were not apparent from 18S rRNA sequences, which are less sensitive to population level variations. Furthermore, we found strong genetic structure between two recent outbreaks, indicating that not all *T. equi* outbreaks can be traced to any single ranch. Although 18S rRNA pinpointed a limited source of introduction, additional resolution using microsatellites indicated that very few samples were in fact identical. This marker system will be useful to help understand the epidemiology of any additional domestic and international *T. equi* outbreaks.

## Competing interests

The authors declare that they have no competing interests.

## Authors’ contributions

CH performed the sequence alignments, participated in the molecular genetic studies, statistical analysis and drafted the manuscript. JB conceived the study, contributed with statistical analysis and drafting the manuscript. GS conceived the study, provided DNA samples and critically revised the manuscript. KP helped with primer design and laboratory experiments. MU provided DNA and scientific guidance. LK provided a whole genome sequence pivotal to this study. DW conceived the study and provided substantial scientific guidance. All authors read and approved the final manuscript.

## Authors’ information

Nucleotide sequence data reported in this paper are available in the GenBank™ database under the accession numbers JX177670, JX177671, JX177672, JX177673.

## Supplementary Material

Additional file 1: Table S1 GenBank entries used for primer design for sequencing the 18S rRNA gene of *Babesia* spp. Click here for file

Additional file 2: Table S2 Eighteen microsatellite *T. equi* loci with primer sequences and amplification conditions. Click here for file
