# Supplementary material for: Genetic characterization of Theileria equi infecting horses in North America: evidence for a limited source of U.S. introductions
Source: Parasit Vectors. 2013 Feb 11;6:35. doi: 10.1186/1756-3305-6-35 (PMC3606381; doi:10.1186/1756-3305-6-35)
Supplement: Additional file 1: Table S1 — GenBank entries used for primer design for sequencing the 18S rRNA gene of Babesia spp. [file 1756-3305-6-35-S1.docx]

**Additional file 1: Table S1**

| Species | Accession number |
| --- | --- |
| *Babesia equi* | Z15105 |
| *Babesia gibsoni* | EU430492 |
| *Babesia major* | GU194290 |
| *Babesia occultans* | HQ331479 |
| *Babesia bigemina* | X59604 |
| *Babesia bigemina* | FJ426361 |
| *Babesia bigemina* | AY648884 |
| *Babesia bigemina* | X59607 |
| *Babesia bigemina* | DQ785311 |
| *Babesia bigemina* | HQ688689 |
| *Babesia bigemina* | FJ869905 |
| *Babesia bigemina* | HQ1977400 |
| *Babesia bigemina* | HQ264118 |
| *Babesia bigemina* | DQ785311 |
| *Babesia bigemina* | EF458196 |
| *Babesia bigemina* | FJ426361 |
| *Babesia bigemina* | EF458206 |
| *Babesia bigemina* | AY603402 |
| *Babesia bovis* | EU407240 |
| *Babesia bovis* | M87566 |
| *Babesia bovis* | HQ264110 |
| *Babesia bovis* | HQ264127 |
| *Babesia bovis* | HM585429 |
| *Babesia bovis* | GQ304525 |

GenBank entries used for primer design for sequencing the 18S rRNA gene of *Babesia* spp.
