# Supplementary material for: Genetic characterization of Theileria equi infecting horses in North America: evidence for a limited source of U.S. introductions
Source: Parasit Vectors. 2013 Feb 11;6:35. doi: 10.1186/1756-3305-6-35 (PMC3606381; doi:10.1186/1756-3305-6-35)
Supplement: Additional file 2: Table S2 — Eighteen microsatellite T. equi loci with primer sequences and amplification conditions. [file 1756-3305-6-35-S2.docx]

**Additional file 2: Table S2**

Eighteen microsatellite *T. equi* loci with primer sequences and amplification conditions.

All PCRs used in this study had the same conditions except for the volume of primers and template. Thermocycler conditions were the same for all PCRs except for the number of cycles. A single PCR consisted of one round of PCR with the heminested labeled forward primer and common reverse primer. Single PCRs were used for both multiplex and singleplex reactions. A single PCR totaled 10μL including 1 μL of 10x buffer, 2 mM MgCl_2_, 0.2 mM dNTPs, 0.4 μM of a labeled-F and R primer (multiplex primer concentrations listed in Table below), 1.0 unit Platinum® Taq (Invitrogen, Grand Island, NY) and 2 μL of template (~5 ng/μL). A fully heminested PCR consisted of two rounds of PCRs, first a primary PCR and then a heminested secondary PCR. The primary heminested PCR had the same conditions as above except as follows: 0.2 μM of each primer (primary-F and R) and 2 μL of template. The secondary heminested reaction also used the same components as above except: 0.8 μM of each primer (labeled-F and R) and 1 μL of primary PCR template. Cycle conditions for secondary heminested PCRs consisted of 10 min, 95˚C; (20 sec, 94˚C; 15 sec, 60˚C; 30 sec, 72˚C) x 35 cycles for a single PCR (singleplex or multiplex), x 25 cycles for primary PCR, or x 20 cycles for secondary PCR; 5 min, 72˚C; held, 16˚C.

| Primer name ^a^ | Primer sequence 5' → 3' | Label | Multiplex mix (primer concentration in μM) | Chromosome | Repeat | Product | No. of alleles identified | % mixed inf. |
| --- | --- | --- | --- | --- | --- | --- | --- | --- |
| CHR1-01-F-primary | CAATGGCGGATAACGAACGG |  |  |  |  |  |  |  |
| CHR1-01-F3 | TTCCTGGACAGTAATGGTAAGTGAT | FAM | 5 (0.6) | 1 | (CTT)_10_ | 260 | 8 | 26.2 |
| CHR1-01-R3 | CTAAGACAACGTTCTGAACATGAATC |  |  |  |  |  |  |  |
| CHR1-05-F-primary | TGTATTGCCGGAATTGGGAGTAG |  |  |  |  |  |  |  |
| CHR1-05-F3 | ATGTGCCTATAACGATTGACTTGAA | FAM | 5 (0.8) | 1 | (TTC)_11_ | 557 | 6 | 9.5 |
| CHR1-05-R3 | TTGCAAACTCAGTCTCGACCAT |  |  |  |  |  |  |  |
| CHR1-11-F-primary | AGCTACCTTATTTTGTGAACTTAGAACATCC |  |  |  |  |  |  |  |
| CHR1-11-F | ATCCCGAAGACGAAAATGTATCTAT | NED | 1 (0.6) | 1 | (GAA)_5_ | 250 | 4 | 21.4 |
| CHR1-11-R | TCCAATATAGACCATCCATTCTCAAAT |  |  |  |  |  |  |  |
| CHR1-14-F-primary_2 | ACTCATCCACCCCTAAAATGTCAACT |  |  |  |  |  |  |  |
| CHR1-14-F | CCAGAATACTGAGCGAGCTGTGAG | VIC | 3 (0.4) | 1 | (AAG)_9_ | 253 | 6 | 11.9 |
| CHR1-14-R2 | AATGCCAATCTGTGGAGCAAATG |  |  |  |  |  |  |  |
| CHR2-01-F2-primary | TGTGTCACTTGGAATACTAGGAGGC |  |  |  |  |  |  |  |
| CHR2-01-F2 | ATGCTACTTCAGGAATGACAGGG | VIC | Single (n/a) | 2 | (GAA)_7_ | 345 | 4 | 0 |
| CHR2-01-R2 | GGTAGTCTGTTTCCATTCTCCACCACTCT |  |  |  |  |  |  |  |
| CHR2-02-F-primary | ATTAGGATGTGTTTCATTGGTAATGGT |  |  |  |  |  |  |  |
| CHR2-02-F | TATAAAGGAAGCGCCAAGTCTCCA | NED | Single (n/a) | 2 | (AAAG)_11_ | 268 | 7 | 21.4 |
| CHR2-02-R | TGGACTTGGCACAAATACAAACGA |  |  |  |  |  |  |  |
| CHR2-07-F-primary | GCGCAGTTATTTCAAGTCGTCAGC |  |  |  |  |  |  |  |
| CHR2-07-F | AAGCCATTGAGGGAAGAAATAGGC | FAM | 4 (0.8) | 2 | (ACT)_7_ | 308 | 3 | 9.5 |
| CHR2-07-R | GGAAACACATTGACTAACAGCTTCCA |  |  |  |  |  |  |  |
| CHR2-09-F-primary | ATGGAGACCCCTGGGTTAGACA |  |  |  |  |  |  |  |
| CHR2-09-F | CTTGCGGACATTTTATTTGCGACT | VIC | 4 (0.2) | 2 | (CTA)_5_ | 255 | 4 | 16.7 |
| CHR2-09-R | TGGTAAAGAGTTCAAAATTGCCCT |  |  |  |  |  |  |  |
| CHR2-11-F-primary | CCACGCAGTTGCAAGACCAATAA |  |  |  |  |  |  |  |
| CHR2-11-F | ATCACTATACCATTGGATCCGTGC | FAM | 5 (0.2) | 2 | (CTAT)_4_ | 203 | 3 | 21.4 |
| CHR2-11-R | CCGTCTTCCCTGAGTTCCGTA |  |  |  |  |  |  |  |
| CHR3 02-F-primary | CCACTGTATATTATGATGGGGAGACAGC |  |  |  |  |  |  |  |
| CHR3 02-F | TGTGGATGGTAAATCGTTTGATCG | NED | 5 (0.2) | 3 | (GCTCCT)_4_ | 308 | 3 | 14.3 |
| CHR3 02-R | CCTCAGTAAGTCCATTCGCAGGTT |  |  |  |  |  |  |  |
| CHR3-05-F2-primary | AACCTATCATATCATAGCAATGCCAA |  |  |  |  |  |  |  |
| CHR3-05-F2 | ATAGCATAGGTGCGATAACATAGAG | PET | Single (n/a) | 3 | (AT)_12_ | 299 | 4 | 9.5 |
| CHR3-05-R2 | ACGATGTTCCTTCAAGCAAACACTA |  |  |  |  |  |  |  |
| CHR3-10-F-primary | TACTCGATGGTATTTCTGATCTGGAGG |  |  |  |  |  |  |  |
| CHR3-10-F | TCTGTTTCGGACTTGCTAAAGGAT | VIC | Single-plex added to Mix 4 | 3 | (AT)_8_ | 310 | 6 | 26.2 |
| CHR3-10-R | TGTTTCACTAATTTCAATTGGTCGTT |  |  |  |  |  |  |  |
| CHR3-11-F-primary | GGAGTAAACTCAAAGAAGCACTAACAG |  |  |  |  |  |  |  |
| CHR3-11-F | TCCTTCTACTCCTCCTGGACCTC | FAM | 3 (0.4) | 3 | (AAC)_4_ | 298 | 2 | 2.4 |
| CHR3-11-R | TCCATCAGGGTCTCCTTCAGC |  |  |  |  |  |  |  |
| CHR3-12-F-primary | CATAAGTTGGGGATGTATTTGGGAC |  |  |  |  |  |  |  |
| CHR3-12-F | ACCTGCCTGCTCTGAAAACAAATC | PET | 5 (0.2) | 3 | (AT)_7_ | 241 | 3 | 0 |
| CHR3-12-R | TCCTGGCATATCCTTGGCAATAAC |  |  |  |  |  |  |  |
| CHR4-07-F-primary | CCTATTGTACAACTATCTTCGGGAGTATGAG |  |  |  |  |  |  |  |
| CHR4-07-F | TACCCTCTGGGTCAGGAAACCAT | VIC | 1 (0.4) | 4 | (CT)_7_ | 296 | 3 | 7.1 |
| CHR4-07-R | CGCAATGACGGATTGATCTACAAG |  |  |  |  |  |  |  |
| CHR4-15-F-primary | GGAGTGAATAAAGATATAGATGACATCGCATG |  |  |  |  |  |  |  |
| CHR4-15-F | TCAGCACATGCGATATGCTCAGTA | FAM | 4 (0.4) | 4 | (AT)_10_ | 252 | 4 | 7.1 |
| CHR4-15-R | AAAACAGCCATCATTACGCAATCC |  |  |  |  |  |  |  |
| UM-03-F-primary | ACCATCACATAGAGCTTCTAGAACACCAG |  |  |  |  |  |  |  |
| UM-03-F | AGAGTCACGTCCAAAACGACCTTC | PET | 3 (0.4) | UM | (AAG)_11_ | 276 | 4 | 16.7 |
| UM-03-R2 | CTCACTAACTGGACTATAACAACATTACG |  |  |  |  |  |  |  |
| UM-09-F-primary_3 | TCACCCTTGACTCACTGGTCGTCTTA |  |  |  |  |  |  |  |
| UM-09-F2 | TATGCGGCATTGATCGTGGAC | FAM | 1 (0.4) | UM | (AGT)_5_ | 265 | 5 | 11.9 |
| UM-09-R3 | GGTACCAAACGTTGTGGATTATCC |  |  |  |  |  |  |  |

UM = Marker is from an unjoined contig most likely part of chromosome 4 (Kappmeyer et al., 2012).

^a^ All "primary" primers are external and all "F" primers are internal. No linkage disequilibrium detected in any pairwise combination using FSTAT. Markers (*n*=7) that worked for Te0044 (18S Group C); CHR 1-01, 1-14, 2-01, 2-02, 2-07, 2-11, 3-11.
